# Histological studies on the marsupium of two terrestrial isopods (Crustacea, Isopoda, Oniscidea)

**DOI:** 10.3897/zookeys.515.9401

**Published:** 2015-07-30

**Authors:** Diána Csonka, Katalin Halasy, Elisabeth Hornung

**Affiliations:** 1 Institute for Biology, Faculty of Veterinary Science, Szent István University, Rottenbiller str. 50, H-1077 Budapest, Hungary; 2 Department of Anatomy and Histology, Faculty of Veterinary Science, Szent István University, István str. 2, H-1078 Budapest, Hungary

**Keywords:** Oniscidea, oostegite, cotyledon, light microscopy, electron microscopy, eco-morphological type

## Abstract

The marsupium, a brood pouch in peracarid crustaceans (Crustacea, Malacostraca) has evolved in terrestrial environment for providing nutrition and optimal conditions for embryogenesis. In the present study we give details on the histology and ultrastructure of its constituting elements such as oostegites and cotyledons. Marsupia of two different eco-morphological types of woodlice, namely the non-conglobating species *Trachelipus rathkii* Brandt, 1833 and the conglobating species *Cylisticus convexus* De Geer, 1778 were investigated. Light microscopic (LM) studies showed some differences in the main structure of the two species’ brood pouch: in *Trachelipus rathkii*, a ‘clinger’ type woodlice, the oostegites bend outwards during brood incubation as growing offspring require more space, while in *Cylisticus convexus*, a ‘roller’ type isopod, the sternites arch into the body cavity to ensure space for developing offspring and still allowing conglobation of the gravid females. The quantitative analysis of the oostegites’ cuticle proved that the outer part is about 2.5 - 3 times thicker compared to the inner part in both species. Electron microscopic (TEM) examinations show only small histological differences in the oostegites and cotyledon structure of the two species. Cellular elements and moderately electron dense fleecy precipitate are found in the hemolymph space between the two cuticles of oostegites. The cells contain PAS positive polysaccharide areas. TEM studies revealed some differences in the cotyledon ultrastructure of the two species. Cotyledons of *Trachelipus rathkii* consist of cells with cristate mitochondria and granular endoplasmic reticulum with cisterns. Cotyledons of *Cylisticus convexus* consist of cells with densely cristate mitochondria and ribosomes attached to vesicular membrane structures. In both species cells with electron dense bodies were observed. We conclude that - besides the differences in marsupial shapes - the fine structure of the oostegites and cotyledons is hardly affected by the eco-morphological type, specifically the conglobating or non-conglobating character of the studied species.

## Introduction

During evolutionary land adaptation Oniscidea have developed various morphological, physiological and behavioral solutions to cope with the challenges of terrestrial life (e.g. desiccation, respiration and reproduction), such as pleopodal lungs, water-resistant cuticle and water conducting system. Concerning reproduction they show an extended parental care (XPC), which is a widespread phenomenon in crustaceans. In the majority of peracarid taxa with XPC, offspring are carried in the female’s body, developing from egg to manca larval stage in a temporal brood pouch (marsupium) (Thiel 2003). Care for late developmental stages appears to be an important adaptation in terrestrial environment.

The brood pouch originally evolved for mechanical protection of eggs and developing embryos under water conditions (Steele 1991). In terrestrial environment the ovigerous females produce a microenvironment in the marsupium, providing fluid and oxygen for the developing young (Hoese 1984, Hornung 2011, Linsenmair 1989, Warburg 1987, Warburg and Rosenberg 1996). The brood pouch is formed during the parturial molt (Suzuki and Yamasaki 1989, Suzuki 2002). Hoese (1984) described two types of the Oniscidean marsupium: the amphibian type and the terrestrial one. In the more primitive amphibian type, the marsupium is open both anteriorly and posteriorly, similarly to the aquatic type, and it is connected to a water-conducting system. Fluid circulates in the water-conducting system, also passing through the marsupium. In the terrestrial type, the brood pouch is not connected to the water-conducting system; however the marsupial cavity is filled up with fluid.

Five pairs of oostegites cover the marsupium, which is tightly sealed ventrally and laterally. Oostegites are leaf-like, overlapping appendages, basally fused with the pereomeres (Hoese 1984, Hoese and Janssen 1989, Suzuki and Yamakasi 1991). Suzuki and Yamakasi (1991) concluded that oostegite formation is controlled by ovarian processes. The factor that stimulates oostegite formation may be the same that regulates vitellogenin synthesis.

The inner structure of the marsupium also differs among woodlice, depending on the phylogenetic position of the species. In some - more developed - species it is divided by segmental cotyledons, which are responsible for nutrition and oxygenation of the offspring (Akahira 1956, Hoese and Janssen 1989, Hornung 2011). Cotyledons are metameric outgrowths on thoracic segments 1-5, which develop only during the marsupial period from transverse ridges of the ventral epidermis. Their shapes and dimensions vary in different species and with the stage of the marsupial period (Hoese and Janssen 1989). Vandel (1925, 1942) recognized that Ligiidae, Trichoniscidae, and Tylidae never possess cotyledons, whereas Oniscidae, Porcellionidae, and Armadillidiidae always do. Lewis (1991) hypothesized that the number of cotyledons is related to both phylogenetic position and habitat characteristics (e.g. drought). She found cotyledon numbers ranging from 4 to 28 per female, investigating several species.

Warburg and Rosenberg (1996) reported on a special structure in the conglobating Mediterranean species, *Armadillo officinalis* and *Schizidium tiberianum*. They found sacs inside the marsupial cavity connected to the marsupial roof. These sacs contained the developing eggs, embryos and mancas organized in small groups.

In species belonging to the ‘roller’ eco-morphological type (Schmalfuss 1984) the oostegites bend only slightly which allows ovigerous females still to conglobate. According to the stereo-microscopic studies of Appel et al. (2011) in such cases sternites arch into the body cavity to provide more space for the developing embryos. They studied the conglobating *Armadillidium nasatum*, *Armadillidium vulgare*, *Pudeoniscus birabeni*, *Circoniscus gaigei*, *Cubaris murina* and the non-conglobating *Neotroponiscus daguerri* and *Neotroponiscus carolii*. Conglobation leads to a displacement and compression of the female’s internal organs, which may cause females to cease feeding in advanced gravidity stages. In non-conglobating (‘clinger’, ‘runner’) species the oostegites bend outwards during brood incubation.

The objective of the present paper was to compare the brood pouches of two basically different eco-morphological types (Schmalfuss 1984), by light- and electron microscopical techniques (LM, TEM).

## Materials and methods

### Examined species

The two investigated species were the non-conglobating ’clinger’ type *Trachelipus rathkii* Brandt, 1833 and the conglobating ’roller’ type *Cylisticus convexus* De Geer, 1778. According to Schmidt (2008) both species belong to the group of the Crinocheta, which is one of the five principal lineages of the Oniscidea. While *Trachelipus rathkii* is a member of the “Trachelipodidae”, *Cylisticus convexus* belongs to the “Cylisticidae” group.

The ovigerous females of the examined species were hand collected in a deciduous forest (*Querco petraeae* – *Carpinetum*) of the Buda-mountains, near Budapest, Hungary, during their reproductive period (from May to June) in 2014.

### Light microscopy

For light microscopic investigations (LM) two nearly same sized ovigerous females per species, in the identical marsupial stage, were fixed in an aqueous solution containing 4 % paraformaldehyde, 2 % glutaraldehyde and 0.1 M phosphate buffer (PB) for 48 hours, followed by rinsing in PB. After fixation, tissues were postfixed in 2% osmium tetroxide in 0.1 M PB for 6 hours. The samples were dehydrated through a graded series of ethanol (50% – 30 min, 70% – 3 h, 90% – 1 h, 100% – 1 day). After dehydration the samples were kept in propylene oxide for 1 day, followed by infiltration in propylene oxide : Durcupan resin (1:1) overnight. Samples were infiltrated with Durcupan for 24 hours and embedded afterwards. Histological sections (1 µm) were cut with a Reichert ultramicrotome and stained with toluidine blue. Several samples from the oostegite were stained with periodic acid-Schiff reagent (PAS) to detect polysaccharides such as glycogen in tissues (2 specimen/species, 10 samples/specimen). The sections were photographed with a Leica microscope.

### Transmission electron microscopy

For transmission electron microscopic (TEM) studies two ovigerous females (same size, identical stage) from both species were injected under the tergite with 12.5% glutaraldehyde (in 0.1 M cacodylate buffer). Dissected oostegites and cotyledons with some eggs were fixed in a solution containing 2.5% glutaraldehyde, 2% paraformaldehyde in 0.1 M cacodylate buffer (2 h) and postfixed in 1 % osmium tetroxide and 0.8% potassium ferricyanide. The samples were dehydrated in a graded series of ethanol (30% – 1 h, 50% – 1 h, 70% – 3 h, 90% – 1 h, 100% – 1 day). They were pre-embedded in the mixture of EPON and 100% aceton (1:1). Finally the pieces were embedded in 100 % EPON for 24 hours. Ultrathin sections (60 nm) were cut with a Reichert Ultracut ultramicrotome, studied and photographed with a JEOL 100 C electron microscope.

### Data analysis and statistical methods

To compare and quantify thickness of the oostegites’ outer and inner cuticles, 60 measurements (2 specimen, 3 sections, 10 measurements/section) of each investigated species were taken using the TEM micrographs (ImageJ and MS Excel software) (Csonka et al. 2013). In order to assess the relevancy of the difference in the oostegite outer and inner cuticle thicknesses we performed a one-way ANOVA test followed by a post-hoc Tukey-test on the cross section specific cuticle thickness (R 2.11.1 software).

The schematic drawings about the structural elements were made with the Inkscape vector graphics editor software.

## Results

### Structure of marsupium

**Figure 1.**
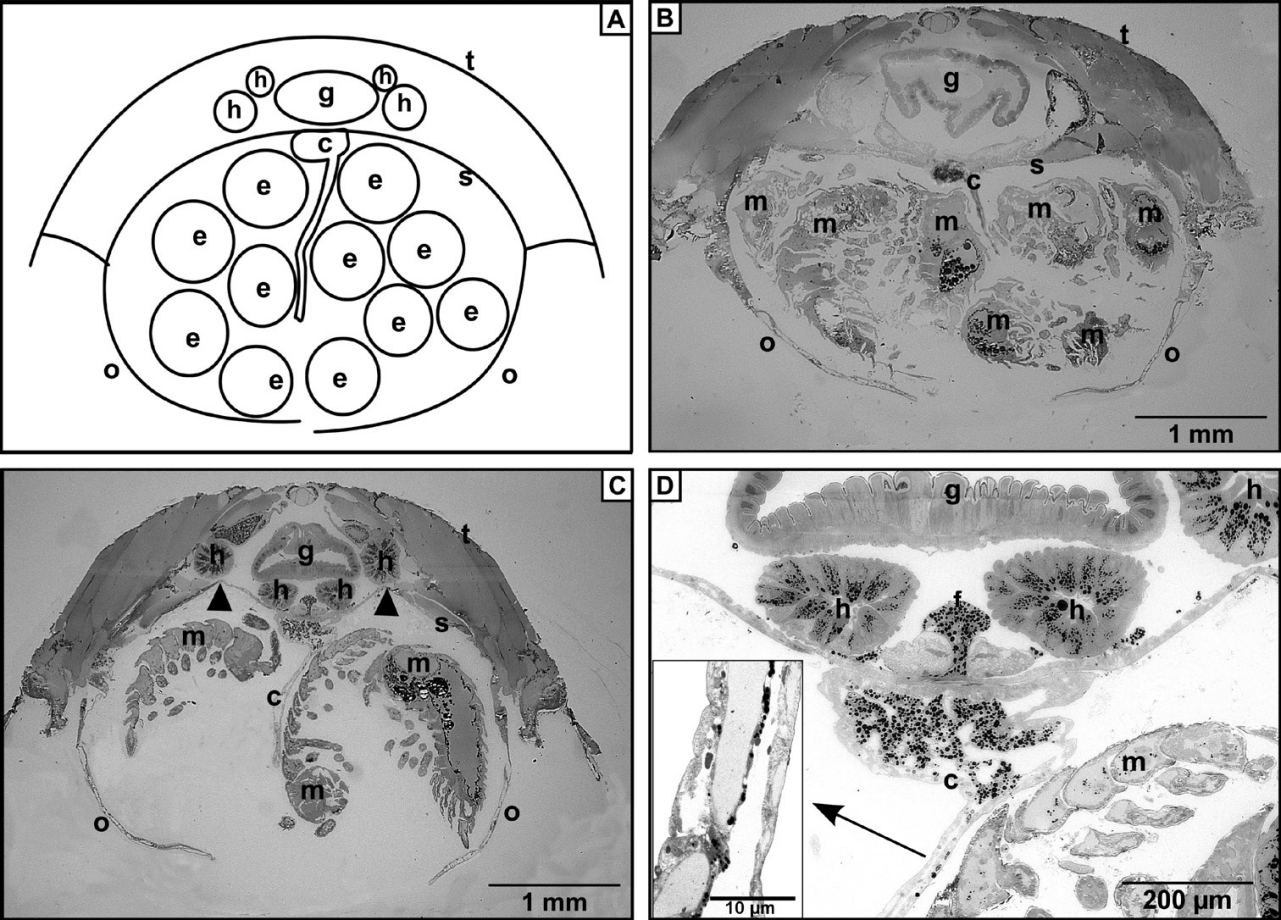
Cross sections of marsupium. **A** Schematic drawing of the brood pouch **B** Marsupium with developing mancas in the non-conglobating *Trachelipus rathkii* **C** Marsupium of conglobating *Cylisticus convexus* in the same stage. Note arching sternites (arrowheads) **D** Higher magnification image of the proximal part of the cotyledon in *Cylisticus convexus*. The cells are filled with darkly stained lipid droplets. Insert: Higher magnification reveals that along the longitudinal axis of cotyledon a beadlike array of lipid droplets lines up. Legends: c – cotyledon, e – egg, f – maternal fat body, g – gut, h – hepatopancreas, m – manca, o – oostegite, s – sternite, t – tergite.

The LM cross sections show several similarities but also some differences between the compared marsupial structures of the two eco-morphological types. In the ‘clinger’ *Trachelipus rathkii* the oostegites bend outwards (Fig. 1A, B). In the ’roller’ *Cylisticus convexus* the sternites arch into the body cavity (Fig. 1C arrow heads). In the cross section of marsupial cavity the developing mancas and cotyledons are clearly recognizable (Fig. 1B, C). Both studied species have one cotyledon situated centrally on each of segments 2-5.

### Structure of oostegite

**Figure 2.**
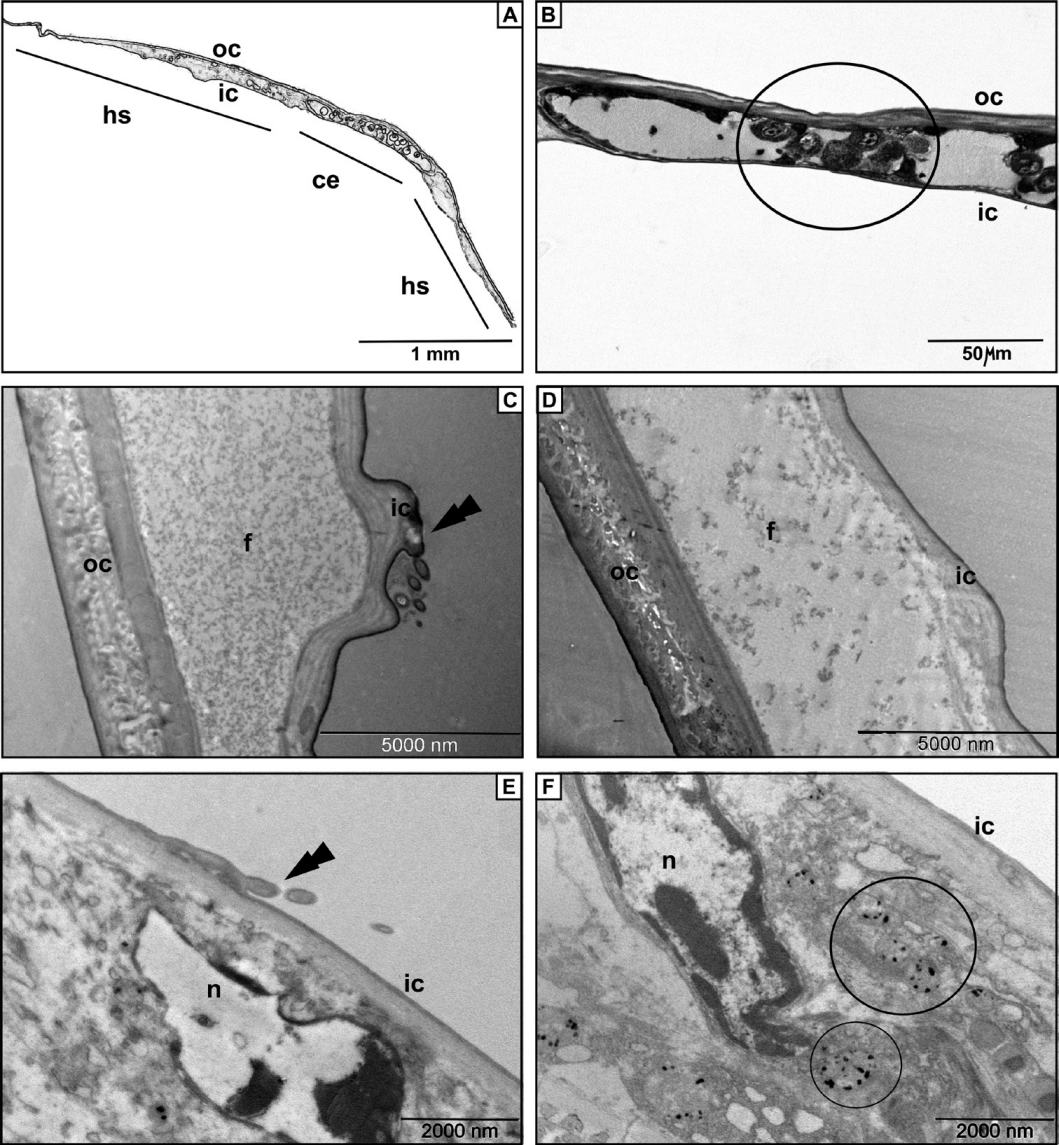
The structure of the oostegite. **A** Schematic drawing of the cross -sectioned oostegite **B** Semithin section after PAS staining with positive cytoplasm in the cells of the oostegite **C** An electron micrograph of the break between cells of *Trachelipus rathkii* oostegite. Note scale-like protrusion of the inner cuticle (arrow) **D** Identical detail in *Cylisticus convexus*. No protrusion was found **E** Cell in the oostegite of *Trachelipus rathkii* below a scale-like protrusion of the inner cuticle (arrow) **F** Cell in the oostegite of *Cylisticus convexus*. Note the membrane-bound electron dense inclusions. Legends: ce – cellular elements, f – fleecy precipitate, hs – hemolymph space, ic – inner cuticle, n – nucleus, oc – outer cuticle.

Both species have five pairs of oostegites (on thoracic segments 1-5), that have the same structure. TEM micrographs show that the outer cuticle of the oostegites is 2.5–3 times thicker compared to the inner one in both species (Fig. 2). We confirmed the morphological observations with quantitative analysis (Table 1). The ANOVA test revealed that the values of cuticle thickness differ significantly when comparing the inner and outer cuticle of the oostegite (p < 0.001).

**Table 1.** The mean thickness (nm) with standard deviation (SD) of the oostegites’ outer (oc) and inner cuticle (ic) layer.

	Mean (oc)	SD (oc)	Mean (ic)	SD (ic)
*Trachelipus rathkii* 1	1828,3	± 233.6	724,76	± 241.3
*Trachelipus rathkii* 2	2001,6	± 183.2	697,5	± 212.3
*Cylisticus convexus* 1	2092,2	± 178.9	671,5	± 102.3
*Cylisticus convexus* 2	1997,3	± 189.6	699,4	± 199.1

In both species the space between the inner and outer cuticle consists of cellular elements and hemolymph space (Fig. 2A, B). This inner structure is similar all along the oostegite. In *Trachelipus rathkii* the hemolymph space contains moderately electron dense fleecy precipitate which is much less pronounced in *Cylisticus convexus* (Fig. 2C, D). The periodic acid-Schiff staining showed PAS positive cytoplasm in the cells within the hemolymph space of oostegite (Fig. 2B). Small scale-like protrusions are recognizable on several electron micrographs of the oostegites’ inner cuticle in *Trachelipus rathkii* (Fig. 2C, E). Similar structures were not observed in the oostegite of *Cylisticus convexus*. In the latter species, membrane-bound electron dense inclusions can be observed in the cells (Fig. 2F).

### Structure of cotyledon

Cotyledons appear in the marsupium among developing offspring in both species. The maternal fat body and the cells of hepatopancreas contain densely stained lipid inclusions, similarly to the proximal part of the cotyledon, whereas along its longitudinal axis these line up in a bead-like array (Fig. 1D).

**Figure 3.**
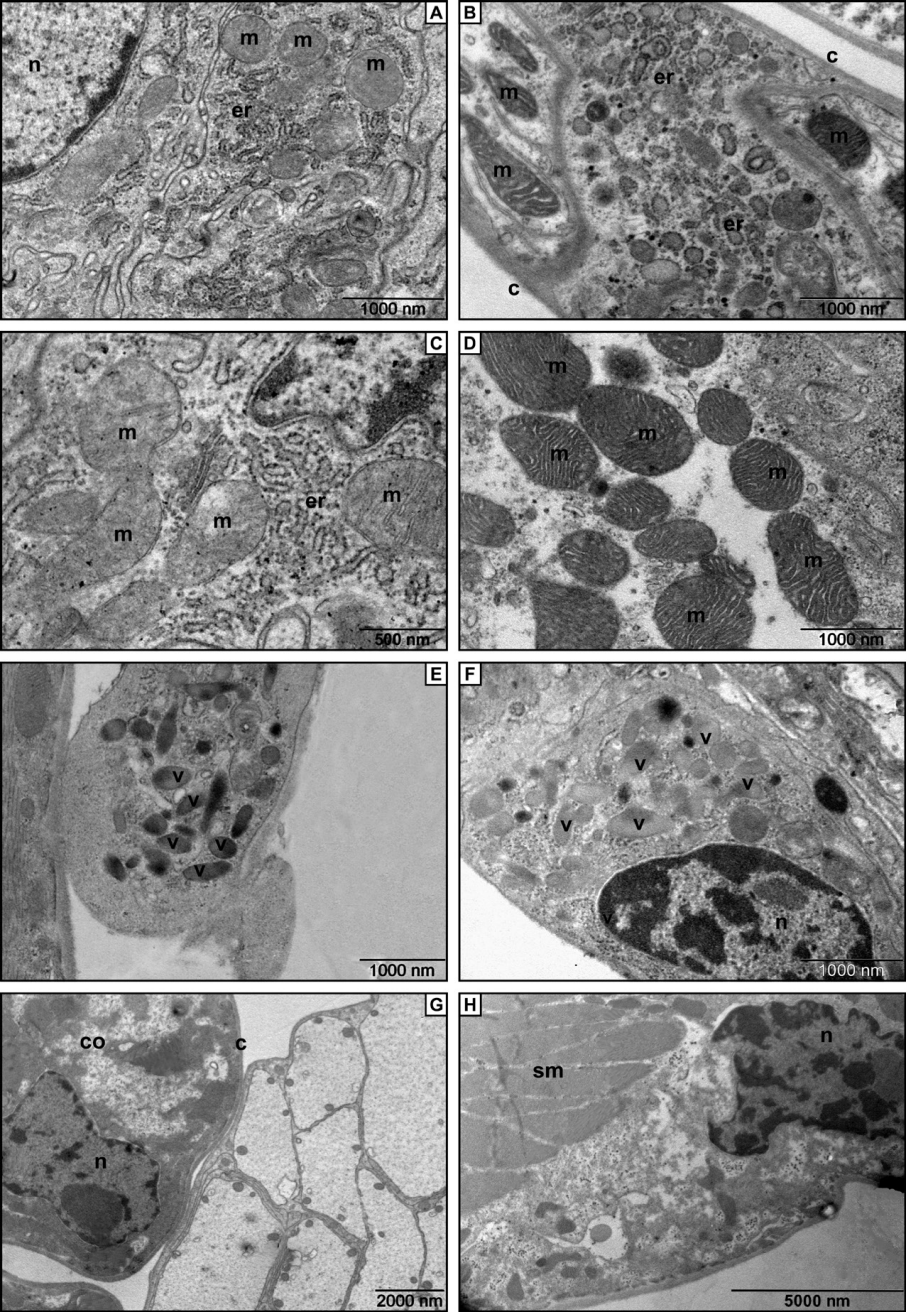
Electron micrographs of the cotyledon. **A** Fine structure of a cell from the medial portion of the cotyledon in *Trachelipus rathkii*. The most abundant cell organelles are mitochondria and rough endoplasmic reticulum **B** In the medial portion of the cotyledon the cells contain vesiculated rough endoplasmic reticulum and mitochondria (*Cylisticus convexus*) **C** Higher magnified detail of the cotyledon in *Trachelipus rathkii*. **D** High power micrograph of the cotyledon in *Cylisticus convexus*. Note the densely cristate mitochondria **E** Rounded ending of the cotyledon with electron dense vesicles (*Trachelipus rathkii*) **F** A cell with large vesicles containing moderately electron dense material (*Cylisticus convexus*) **G** Cotyledon ending of *Trachelipus rathkii* covered by a thin cuticle **H** Bundles of striated muscle fibers located at the base of cotyledon (*Cylisticus convexus*). Legends: c – cuticle, co – cotyledon, er – rough endoplasmic reticulum, m – mitochondria, n – nucleus, sm – striated muscle, v – vesicle.

The electron micrographs (TEM) show cotyledons covered by an extremely thin cuticle (Fig. 3B). In both species these sternite outgrowths are built up by cells, which contain abundant mitochondria and rough endoplasmic reticulum (Fig. 3). We found obvious differences in the two investigated species concerning the structure of the mitochondria and the endoplasmic reticulum. The cotyledons in *Trachelipus rathkii* consist of cells with common cristate mitochondria and rough or granular endoplasmic reticulum with cisterns (Figs 3A, C). On the contrary in *Cylisticus convexus* there were cells with densely cristate mitochondria, their endoplasmic reticulum was dominated by rounded vesicle-like membranes instead of the common cisterns (Figs 3B, D).

We found cells in the cotyledon of both species with cytoplasm mainly characterized by the presence of several electron dense vesicles (Figs 3E, F). At the base of the cotyledon bundles of striated muscle fibers are present in both species (Fig. 3H).

## Discussion

We examined the structure of the marsupium in two different eco-morphological types of woodlice: non-conglobating (*Trachelipus rathkii*) and conglobating (*Cylisticus convexus*). We predicted that differences between the two eco-morphological types are reflected in idiosyncratic morphological features of their brood pouches.

Light microscopic results here concurred with the statements of Appel et al. (2011). They compared the relationship between the morphology of the marsupium and the eco-morphological type with stereo microscopic techniques. By their findings the gravid females of conglobating species have sternites arching into the body cavity to provide more space for developing offspring. Warburg and Rosenberg (1996) recognized that in conglobating *Armadillo officinalis* and *Schizidium tiberianum* developing eggs, embryos and mancas are grouped into marsupial sacs. We did not find sac-like marsupial structure in the roller *Cylisticus convexus*, so that is not related to the conglobating form.

Treviranus and Treviranus (1816) were the first to describe cotyledons and oostegites of terrestrial isopods as parts of the marsupium. They assumed that cotyledons would be necessary for the nutrition of developing young. Patanè (1940, 1951) studied marsupium in *Porcellio laevis*, *Armadillidium cinereum*, *Trichoniscus pusillus provisorius*, *Ligia italica*, *Anilocra physodes* and assumed that marsupial fluid enters the brood pouch via cotyledons and oostegites and that the respiratory function predominates over that of nutrition. He mentioned that the ventral integument of the females is very thin and permeable. This could facilitate exchange of nutrients between body cavity and the marsupium. The outer cuticle of the oostegites has to be thick and impermeable to give protection against desiccation (Hoese 1984). Our quantitative analysis showed that in the two species studied here the outer cuticle is about 2.5–3 times thicker compared to the inner one in both species. In *Porcellio dilatatus* Luca (1965) discovered large secretory cells in the oostegites. According to our observations oostegites consist of cellular elements and hemolymph space. In the hemolymph space we observed moderately electron dense fleecy precipitate in varying amount in both species. This may be the solid part of the hemolymph precipitated during fixation. The PAS positive reaction of the oostegite cell cytoplasm may prove the presence of polysaccharides for the cuticle. Moreover, electron microscopic studies revealed small protrusions on the oostegites’ inner cuticle in *Trachelipus rathkii*. These outgrowths could be engaged with sensory function monitoring the marsupial fluid content, although we failed to detect dendritic processes of neurons in contact with them. In this way they probably represent simply architectural elements like plaques or scales.

Akahira (1956) studied the epithelium and found a mucous mass inside and outside the cotyledons and some blood cells in the marsupium of *Porcellio scaber*. He suggested that cotyledons are storage organs for a mucous mass on which the embryos feed during development. Hoese and Janssen (1989) examined the brood pouch in isopods *Armadillo ausseli*, *Armadillidium vulgare*, *Hemilepistus aphganicus*, *Hemilepistus reaumuri*, *Hyloniscus riparius*, *Philoscia muscorum*, and *Porcellio scaber*. These authors looked for indications of transport mechanisms through the cotyledon epithelium. They found that the histological features of cotyledon integument are identical with those of a locally differentiated and periodically active transport epithelium and it contains a portion of the maternal fat body. Our micrographs show also the maternal fat body, which is a fat-storing adipose tissue enclosing the ventral nerve cord (Hoese and Janssen 1989). Picaud and Souty (1980) and Picaud et al. (1989) detected vitellogenin synthesis in the fat body of *Porcellio dilatatus*. We observed densely stained lipid inclusions at the proximal part of the cotyledon. The structures are present also in the cells of hepatopancreas and the mancas, and resemble lipid droplets (Štrus and Blejec 2001). Our examination showed that cotyledons in both species are built up by cells, which contain different types of mitochondria. The morphology of those is related to the functional states of cells. Mitochondria with tubes and vesicles are characteristic features of steroid producing cells in many species from ciliates to mammals, such as adrenal cortex and Leydig-cells (Secchi and Lecaque 1981, Bloom and Fawcett 1994). It may be that the cotyledon cells studied here are also engaged in the synthesis of similar molecules, although vesicular profiles could not be detected.

It is noteworthy that striated muscle fibers are present at the base of cotyledons. We suppose that they play an important role in the fixation of the cotyledons’ basal part and they allow a certain degree of mobility.

We found only small histological differences in the oostegite and cotyledon structures of the two species with different eco-morphological background. Since both species belong to the same lineage of Oniscidea, these differences probably reflect to the physiological state of the animal, rather than the eco-morphological type. Further investigations are needed to compare several other species with different phylogenetic position in the future to make general statements.

## Conclusions

Our findings show that the gross anatomy of the brood pouch in the examined species’ agrees with that of species studied earlier. The main structure of the oostegites is similar in both species. Small protrusions of the oostegites’ inner cuticle are recognizable only in *Trachelipus rathkii*. In the case of cotyledon the electron micrographs show differences in the two investigated species concerning the structure of the mitochondria and the endoplasmic reticulum, but these features can be related to their physiological state. The proximal part of the cotyledon contains dark vacuoles. These are lipid inclusions which might represent an energy storage site.
